# Unveiling Hidden Dynamics of Hippo Signalling: A Systems Analysis

**DOI:** 10.3390/genes7080044

**Published:** 2016-08-05

**Authors:** Sung-Young Shin, Lan K. Nguyen

**Affiliations:** 1 Department of Biochemistry and Molecular Biology, School of Biomedical Sciences, Monash University, Clayton Victoria 3800, Australia; sungyoung.shin@monash.edu; 2 Biomedicine Discovery Institute, Monash University, Clayton Victoria 3800, Australia

**Keywords:** hippo signaling, ERK MAPK signaling, mathematical modelling, systems analysis, network dynamics, cell fate determination, feedback regulation

## Abstract

The Hippo signalling pathway has recently emerged as an important regulator of cell apoptosis and proliferation with significant implications in human diseases. In mammals, the pathway contains the core kinases MST1/2, which phosphorylate and activate LATS1/2 kinases. The pro-apoptotic function of the MST/LATS signalling axis was previously linked to the Akt and ERK MAPK pathways, demonstrating that the Hippo pathway does not act alone but crosstalks with other signalling pathways to coordinate network dynamics and cellular outcomes. These crosstalks were characterised by a multitude of complex regulatory mechanisms involving competitive protein-protein interactions and phosphorylation mediated feedback loops. However, how these different mechanisms interplay in different cellular contexts to drive the context-specific network dynamics of Hippo-ERK signalling remains elusive. Using mathematical modelling and computational analysis, we uncovered that the Hippo-ERK network can generate highly diverse dynamical profiles that can be clustered into distinct dose-response patterns. For each pattern, we offered mechanistic explanation that defines when and how the observed phenomenon can arise. We demonstrated that Akt displays opposing, dose-dependent functions towards ERK, which are mediated by the balance between the Raf-1/MST2 protein interaction module and the LATS1 mediated feedback regulation. Moreover, Ras displays a multi-functional role and drives biphasic responses of both MST2 and ERK activities; which are critically governed by the competitive protein interaction between MST2 and Raf-1. Our study represents the first in-depth and systematic analysis of the Hippo-ERK network dynamics and provides a concrete foundation for future studies.

## 1. Introduction

Initially identified and named through screens for tumour suppressors in *Drosophila* (fruit flies), the Hippo signalling pathway was found well conserved in mammals and known to play crucial roles in the regulation of cell proliferation, differentiation, survival and programmed cell death [1,2]. The Hippo pathway has sustained immense interest in recent years due to its strong involvement in organ size control and human cancer development [3]. The central components of the Hippo pathway comprise a core kinase cassette consisting of the mammalian Ste20-like kinase 1/2 (MST1/2) and large tumour suppressor kinase 1/2 (LATS1/2); and the downstream transcriptional coactivators Yes-associated protein (YAP) and Transcriptional coactivator with a PDZ-binding domain (TAZ) [4]. MST1/2 phosphorylates and activates LATS1/2, which subsequently phosphorylates YAP and TAZ [5]. The functional activity of the phosphorylated YAP and TAZ is inhibited through nuclear exclusion due to sequestration in the cytoplasm, and/or proteasomal degradation [5].

Recent studies have shown that the Hippo pathway does not function in isolation but is tightly integrated with the Raf/MEK/ERK (ERK pathway in short) and Akt pathways at multiple levels of crosstalk to coordinate cell fate dynamics [6,7]. The first crosstalk layer is through Akt where it phosphorylates MST2 and inhibits its functional activity toward LAST1. In addition, Akt-mediated phosphorylation of MST2 enhances its binding to Raf-1, which interferes with MST2 dimerization and activation, and at the same time suppresses Raf-1/ERK activation by sequestrating Raf-1 away from the Ras/Raf-1 complex [6,8]. The next layer of crosstalk is the LATS1-mediated feedback phosphorylation of Raf-1 on Serine 259. Raf-1 phosphorylated on Serine 259 is inactive towards the ERK pathway, and its dephosphorylation is a central part of the physiological Raf-1 activation process [7,8,9]. On the other hand, Serine 259 phosphorylation promotes Raf-1 binding to MST2 and MST2 inhibition [6,8]. These data together highlight that the Hippo-ERK signalling network is governed by a web of intertwined regulatory mechanisms, and thus the resulting network behaviours can be highly complex, dynamic and rich. “*Can we identify these potential network behaviours?*” and “*When and how do they arise*?” are important questions but not yet examined. It is expected, however, that the precise network dynamics that manifest in a specific cell type (or condition) would be determined by the balance between the governing regulatory mechanisms, which may or may not always be at play and possibly to different extents depending on the specific cellular context.

Using breast cancer cells, we previously showed that increasing Akt activity precipitously inhibited MST2 activation in a switch-like manner, whereas ERK activation was only affected when the LATS1 feedback of inhibitory Serine 259 phosphorylation is operating [6]. Moreover, Ras can activate both the ERK and MST2 pathways, but owing to the Akt mediated MST2 inhibition, Ras inactivates MST2 instead in cells where Akt is strongly activated by Ras [6,7]. However, it is increasingly clear that cells of different tissue origins (or from different patients) may display significant variations in the expression of signalling network components [10,11,12]. Indeed, quantitative abundance of the key nodes of the Hippo-ERK network obtained by mass spectrometry from various tissue specific cell lines showed strong variations in their values [6,7]. As another example, lymphoblastoid cells from a cohort of 95 patients exhibited considerable individual-to-individual protein abundance variations [11]. It is also apparent that protein expression levels can change significantly in cells harbouring genetic alternations such as gene amplification, mutation or epigenetic modifications, compared to normal cells [12]. As variations in expression of the network components can lead to dramatic changes in network behaviours, studying network behaviours in a specific cellular context may not be generalizable to others. Thus, in order to gain a more global and deeper understanding of systems dynamics, it is imperative to study the network in a systematic manner spanning different cellular contexts. Understanding the context-specific dynamic properties of Hippo-ERK signalling and how the different regulatory mechanisms interplay to drive the network dynamics are of considerable importance but have not been explored so far.

In the present work, we employed computational modelling and extensive in silico simulations and analyses to explore the salient dynamic features of Hippo-ERK signalling. We constructed and experimentally trained a mathematical model of the Hippo-ERK network; and used this model to investigate the input-output response profiles of the system under different cellular contexts, through systematic perturbations of model parameters over wide ranges of parameter values. We uncovered that the Hippo-ERK network can generate highly diverse dynamic profiles that can be clustered into several distinct dose-response patterns. For each pattern, we provided the mechanistic explanation underlying the observed phenomenon. Notably, we demonstrated that Akt could trigger diverse ERK activation response patterns in a context-dependent manner. We showed that the Raf-1/MST2 protein interaction interplays with the LATS1 mediated feedback to bring about the dual, opposite roles of Akt in ERK regulation. On the other hand, Ras displays a multi-functional role and drives biphasic responses of both MST2 and ERK activities; which are critically mediated by the competitive protein interaction between MST2 and Raf-1. Our study represents the first systematic analysis of the Hippo-ERK signalling network and provides a concrete foundation for future studies.

## 2. Materials and Methods

The mathematical model incorporates protein interactions, phosphorylation reactions, and feedback regulations between Hippo and ERK pathways, which was described by ODEs. Michaelis-Menten kinetics were largely used to model the phospho-reactions and feedback regulations, and the mass-action kinetics were used to describe association/dissociation of proteins. The modelling is described in details in the Supplementary Materials (Tables S1–S3) where all the model equations and parameters are provided. The model was implemented in MATLAB, which was also used for parameter estimation using genetic algorithm. The Supplementary Materials Table S4 contains the parameter values clustered for the response pattern classification. An SBML version of the model is deposited in the Biomodels database (www.biomodels.com) and also available upon request.

## 3. Results

### 3.1. Mathematical Modelling of the Integrated MST2-ERK Network Dynamics

**Figure 1:**
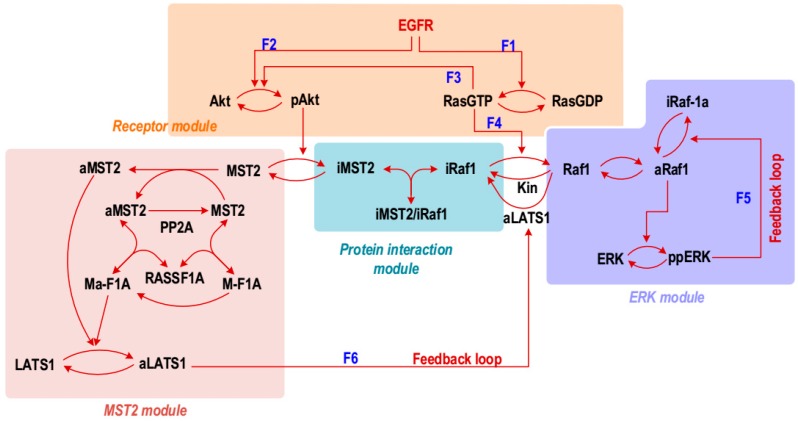
Schematic diagram of the Hippo-ERK network showing the molecular species and the reactant proteins involved in binding and (de)phosphorylation reactions: aRaf1, active Raf-1 phosphorylated at Serine 338; iRaf1, inactive Raf-1 phosphorylated at Serine 259; iMST2, inactive MST2 phosphorylated by Akt; aMST2, active MST2; iMST2/iRaf1, iMST2-iRaf1 complex; M-F1A, RASSF1A-MST2 complex; Ma-F1A, aMST2-RASSF1A complex; aLATS1, active LATS1; ppERK, double-phosphorylated, high-activity ERK; pAkt, phosphorylated, activated Akt; Kin, kinases other than LATS1 phosphorylating Raf-1 on Serine 259. F_1_–F_6_ indicate the crosstalk and feedback regulations of the ERK and MST2 pathways.

To investigate the quantitative and dynamic properties of the integrated MST2-ERK network, we first extended a previously published model [6]. In this model, Akt and Ras were considered as direct inputs into the model as time-dependent functions for simplicity, where the forms of these functions were informed by experimental data. In this study, we extended the network model by introducing the detailed regulatory mechanisms of Ras and Akt activation, and EGFR as a new input node. This extension was necessary to directly calibrate the model against dynamic data of Akt, and enable a systematic investigation of potentially different roles of crosstalk/feedback mechanisms between the ERK and MST2 pathways. The resulting model structure (i.e., reaction scheme) is given in Figure 1, which explicitly describe the mechanistic activation of Akt and Ras following activation of the EGF receptor (EGFR). As such, the new model consists of four sub-modules, as highlighted in Figure 1. (i) The *receptor module* includes Ras and Akt activation triggered by activated EGFR, as well as Ras-induced Akt activation [13,14,15]; (ii) The *ERK module* describes activation of the Raf-1/MEK/ERK MAPK cascade initiated by Ras mediated Raf-1 activation, including the negative feedback loop from ERK to Raf-1 [16]. To reduce the number of model parameters without significantly compromising the network dynamics properties, the MEK and ERK activation steps are lumped into a single step; (iii) The *MST2 module* describes activation of the MST2/LATS signalling cascade, which is mediated by the scaffold protein Ras association domain family 1 isoform A (RASSF1A). Importantly, this module contains the cross-pathway feedback from LATS1 to Raf-1, where active LATS phosphorylates Raf-1 and promotes its complexes formation with MST2, thereby inhibiting Raf-1 activity [6,8]; (iv) The *protein interaction module* describes the non-catalytic binding between MST2 and Raf-1, and that this binding is promoted when MST2 and Raf-1 are phosphorylated by Akt and LATS1, respectively [6,7,8]. This module together with the LATS1 mediated feedback loops constitute critical mechanisms that link the Hippo and ERK pathways in mammalian cells.

**Figure 2:**
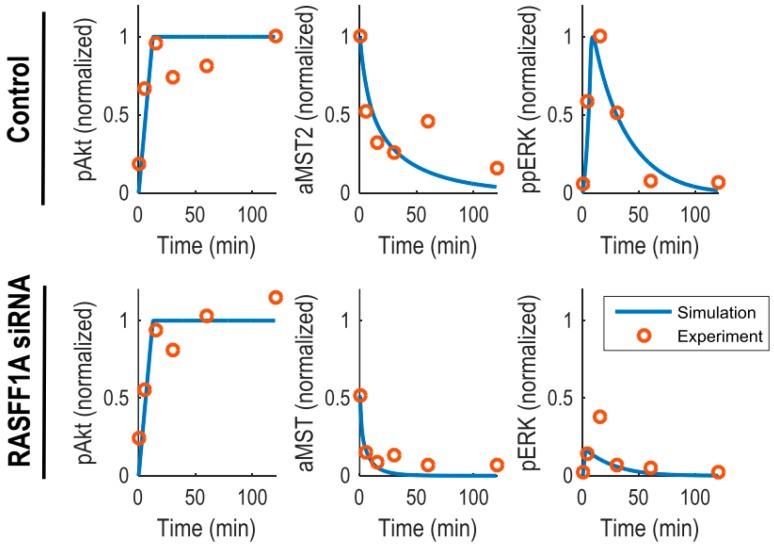
Model training and kinetic parameter estimation for the integrated Hippo-ERK signalling network. The time-course data were obtained in HeLa cells [6]. The levels of phosphorylated (active) Akt (pAkt), ERK (ppERK) and MST2 (aMST2) were measured at 6 time points over 2 h serum stimulation under untreated and RASSF1A knockdown conditions. The experimental and simulation data are represented by the red circle and blue line, respectively. RASFF1A siRNA denotes RASSF1A knockdown conditions.

Thus, the developed model incorporates protein interactions, phosphorylation reactions and feedback loops, which was described by ordinary differential equations (ODEs). The resulting model comprises 20 ODEs and a total of 50 kinetic parameters, described using a combination of mass-action and Michaelis-Menten kinetics (see Supplementary Materials Tables S1 and S2). To generate quantitative predictions, we calibrated the model and estimated kinetic parameters using a set of time-course data obtained from HeLa cells [6]. These include the levels of phosphorylated (active) Akt (pAkt), ERK (ppERK) and MST2 (aMST2) measured at 6 time points over 2 h serum stimulation under untreated and RASSF1A knockdown conditions (Figure 2). The data show that while RASSF1A knockdown did not affect the kinetics of Akt activation, it significantly downregulated the basal and temporal levels of MST2 activity. Similarly, ERK activity were remarkably reduced by downregulation of RASSF1A, however its transient dynamics was retained albeit with a lower peak. For parameter estimation, we used genetic algorithm, a computational method that was inspired by evolutionary biology concepts such as selection, crossover, mutation, etc. [17,18,19]. In engineering fields, this algorithm is widely exploited to find approximate solutions of optimization problems [20]. To ensure the biological relevance of the kinetic parameters during, we constrained the parameters within their respective physiologically plausible ranges before fitting. These ranges were informed by previous studies [16,21,22,23], and also used in our previous work [24,25]. For examples, k_a_ (association rate) = [1 × 10^−4^, 1 × 10^4^] (nM^−1^·min^−1^); k_d_ (dissociation rate) = [1 × 10^−4^, 1 × 10^1^] (min^−1^); k_c_ (catalytic rate) = [1 × 10^−4^, 1 × 10^3^] (min^−1^); V_max_ (max velocity) = [1 × 10^−4^, 1 × 10^3^] (nM·min^−1^) and K_m_ (Michaelis-Menten constant) = [1 × 10^−4^, 1 × 10^4^] (nM). Model simulations of the best fitted parameter set showed that the model quantitatively recapitulates all the observed dynamics of the MST2-ERK network very well (Figure 2).

### 3.2. Diverse Dose-Response Profiles Generated by the Interlinked Hippo-ERK Network

Feedback loops have been long known to generate complex and non-linear systems dynamics [16,22,26,27,28,29]. Our recent work further demonstrates that reversible, non-catalytic protein-protein interaction motifs could also bring about rich repertoire of complex dynamics features to signalling cascades by generating hidden feedback and feed-forward loops [7,30]. Given that the Hippo-ERK network features both these types of design in its wiring, we hypothesize that they can provoke diverse systems dynamics patterns under different cellular contexts. Nevertheless, what these patterns are and when or how they may arise are questions not yet examined.

**Figure 3:**
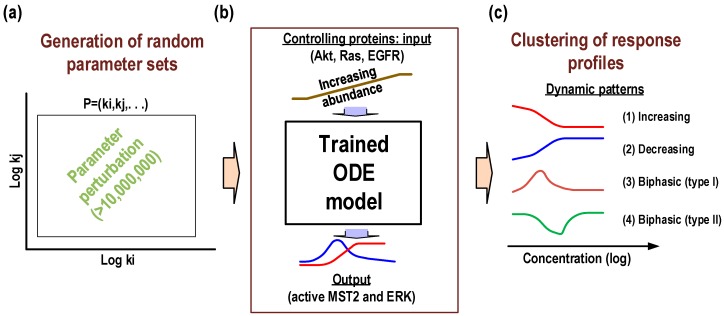
Illustration of the simulation procedure for response pattern clustering. The kinetic parameters underlying six critical regulatory links (denoted F_1_ to F_6_ in Figure 1) are systematically varied within large ranges around their nominal values between 10^−3^ and 10^3^ fold of the fitted values. A large number (>10 million) of parameter sets were generated (**a**); The steady-state response profiles of active MST2 and ERK for each set were simulated to increasing expression of the controlling proteins (i.e., Akt, Ras and EGFR) (**b**); Diverse profile profiles of the Hippo-ERK network are clustered into distinct four different response patterns (**c**).

To address these questions, we carried out extensive computational simulations and analyses using the calibrated model. We focus on the steady-state behaviours of the system by simulating the dose-response profiles (i.e., input-output relationships) of active MST2 (aMST2) and ERK (ppERK), considered as the network’s two major outputs, in response to increasing abundance of specific controlling inputs, in this case of Akt, Ras and EGFR, referred to as the “controlling proteins” (Figure 3). The crosstalk and feedback interactions between MST2 and ERK pathways are expected to play important roles in determining the dynamic behaviours of the overall network. Thus, to explore possible dose-response patterns we selected six critical regulatory links (denoted F_1_ to F_6_ in Figure 1) that mediate the crosstalk and feedback regulations between the pathways, and varied the kinetic parameters underlying these six links randomly and in combination within large ranges around their nominal values (i.e., between 10^−3^ and 10^3^ fold of the fitted values, Figure 3). Note that the remaining parameters were fixed at their fitted values. A total of >10 million parameter sets were generated, for each set the described dose-response profiles were simulated. While network topology is likely conserved between cell types or tissues, the strength of network connections may strongly vary between different molecular contexts [11,12,31]. The extensive parameter variation introduced in our simulations is thus essential to ensure we fully capture the dynamics repertoire of the network that may occur under various molecular backgrounds.

**Figure 4:**
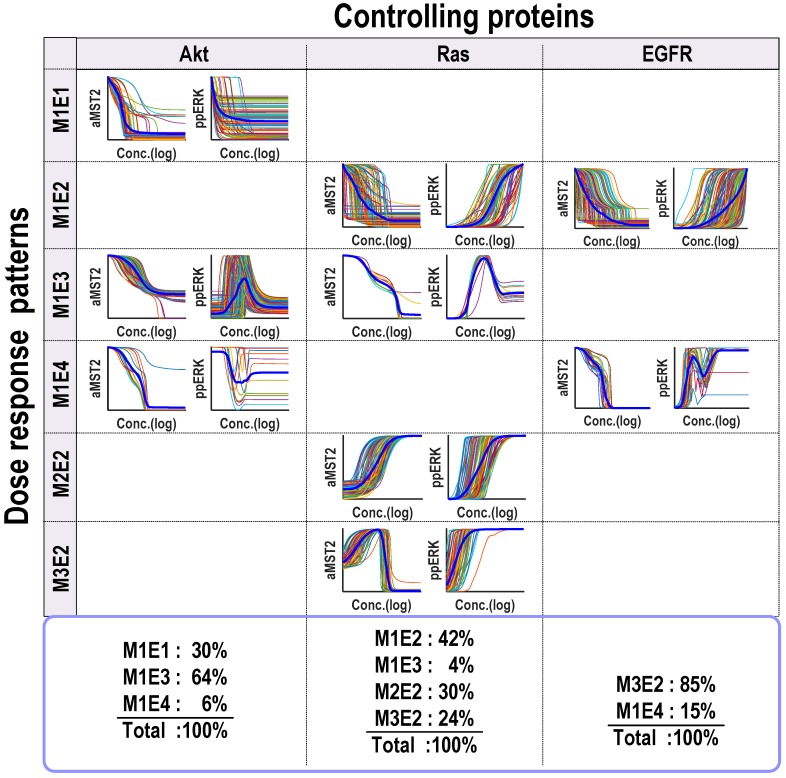
Clustering the dose-response profiles into four dynamic patterns. Combining two network outputs (active MST2 and ERK), the Hippo-ERK network generates a total of eight different patterns for the three controlling proteins (Akt, Ras and EGFR). The *x*-axis of each subplot is the log concentration of a controlling protein and the *y*-axis is the normalized activity of aMST (ppERK) by its maximum.

Model simulations showed that the Hippo-ERK network could generate diverse response profiles, which can be clustered into several distinct dose-response patterns for both the outputs, active MST2 and ERK (Figure 4). Specifically, four different dose-response patterns that occur frequently were identified: (1) increasing; (2) decreasing; (3) biphasic type I and (4) biphasic type II. Under these definition, patterns 1 (or 2) represents a monotonic increase (or decrease) of active levels of the output to increasing abundance of the controlling input protein; while patterns 3 (or 4) describes a biphasic response of the output to increasing abundance of the controlling protein in a concave-down (or concave-up) manner. Combining both network outputs (active MST2 and ERK), we found that the Hippo-ERK network can generate a total of 8 different patterns for the three controlling proteins (Akt, Ras and EGFR). Shown in Figure 4, Akt can trigger 3 distinct combined patterns (denoted M_1_E_1_, M_1_E_3_ and M_1_E_4_) as the regulating protein; while Ras can generate 4 patterns (M_1_E_2_, M_1_E_3_, M_2_E_2_ and M_3_E_2_) and EGFR produces 2 patterns (M_1_E_2_ and M_1_E_4_). Here, the notation “M*_i_*E*_j_*” means active MST2 (“M”) follows pattern *i* and active ERK (“E”) follows pattern *j* as defined previously (subscripts *i* and *j* can range between 1 and 4).

Although in principle each controlling protein can generate up to 16 combinatorial dose-response patterns for MST2 and ERK on the basis of the 4 defined patterns, the fact that only a limited subset of these are actually found indicates that the systems dynamics are well constrained by the network wiring. At the same time, since more than one patterns are observed for each controlling proteins, the Hippo-ERK systems dynamics also display robust plasticity determined by the network’s kinetic parameters. These observations confirm the view that systems dynamics are a product of both network topology and network parameterization [32,33].

### 3.3. Akt Generates Diverse ERK Response Patterns

Our model simulations previously showed that Akt can generate three distinct response patterns, namely M_1_E_1_, M_1_E_3_ and M_1_E_4_ (Figure 4, first column). Interestingly, while Akt always decreases MST2 activity, it induces diverse response profiles of ERK activity, provoking a decreasing ERK activity response in some case, and triggering biphasic ERK activity responses in others. In fact, M_1_E_3_ is the most frequently observed pattern, accounting for 64% of the total sampled parameter sets, followed by M_1_E_1_ (30%) and M_1_E_4_ (4%). Here, we seek to understand how Akt can generate such different dynamic behaviours and aim to provide a mechanistic explanation of the underlying mechanisms in each case.

**Figure 5:**
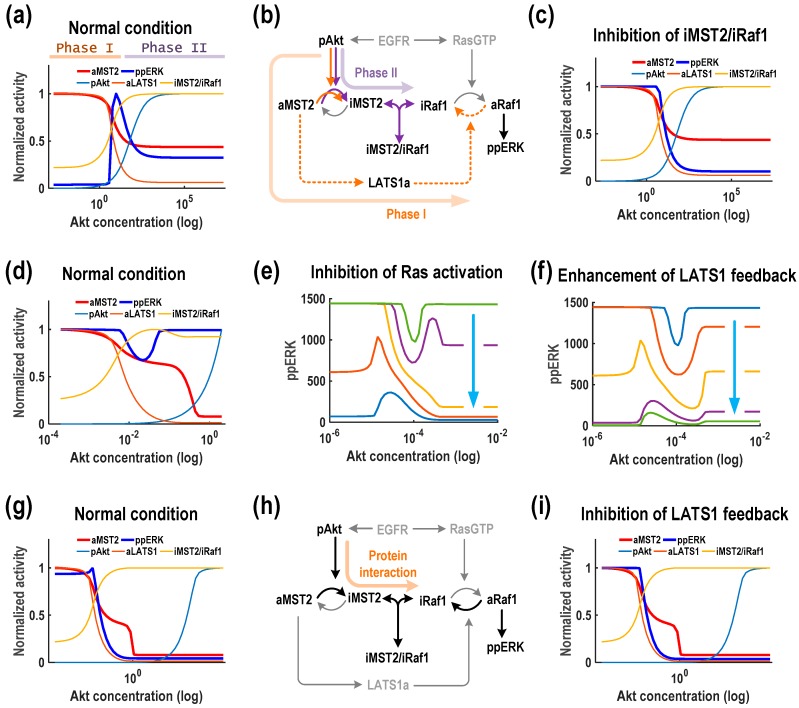
Akt generates diverse ERK response patterns. (**a**–**c**) The pattern M_1_E_3_ where Akt downregulates MST2 activity and induces a biphasic (concave-down) response of ERK activity; (**d**–**f**) The pattern M_1_E_4_ where ERK activity displays the type II biphasic (concave-up) response against increasing Akt; (**g**–**i**) The pattern M_1_E_1_ in which Akt consistently inhibits both ERK and MST2 activation. Note that the response profiles to Akt were generated using a representative parameter set that show the same patterns as in Figure 4 (see Supplementary Materials Table S4).

We first consider the most common pattern M_1_E_3_, where Akt induces a biphasic (concave-down) response of ERK activity. This pattern indicates that Akt could play opposing roles in regulating ERK activity which switch in a dose-dependent manner: Akt functions as a promoter when its levels are low, but as an inhibitor when its levels are high. To investigate the underlying mechanisms of this biphasic profile, we simulated the steady-state levels of key network components in response to increasing Akt, as shown in Figure 5a. Analysing these curves and the network wiring suggests that ERK’s biphasic profile can be generated mechanistically through two different phases as the balance of network regulation mediated by the LATS1 mediated feedback and MST2-Raf-1 interaction tips. Specifically, increasing Akt starting at a low level phosphorylates and inhibits MST2, thus relieving the LATS1-to-Raf-1 negative feedback, leading to increased Raf-1/ERK activity (Phase I). However, further increase in Akt promotes accumulation of the inactive form of MST2 which sequesters Raf-1 from being activated by Ras via formation of the MST2-ERK complexes, thus resulting in suppressed ERK activity at high Akt levels (Phase II, indicated in Figure 5b). To confirm that this mechanism indeed underlies M_1_E_3_, we first blocked the protein interaction module by setting the association rate between MST2 and Raf-1 to null. Consequently, the biphasic ERK activity profile was abolished and replaced by a monotonic decreasing pattern; whereas the activity profile MST2 was not affected (Figure 5c). Next, we inhibited the LATS1 feedback loop and found that the ERK activity profile was replaced by a monotonic increasing pattern (Supplementary Materials Figure S1a). Together, these simulations support that the Raf-1/MST2 interaction module interplays with the LATS1 mediated feedback to bring about the dual, opposite roles of Akt in ERK regulation.

Another intriguing pattern identified in our simulations was M_1_E_4_ where ERK activity displays the type II biphasic (concave-up) response against increasing Akt. In contrast to the previous case, here Akt acts as an inhibitor of ERK at low levels, but as a facilitator at high levels (Figure 5d). ERK activity is high at both low and high Akt levels, which suggests that Raf-1 is also highly active at these concentrations. Raf-1 activation is controlled by Ras and LATS1 as opposing regulators: Ras-induced phosphorylation at Serine 338 activates Raf-1 but LATS1-induced phosphorylation at Serine 259 inhibits it. Thus, at a lower concentration, Akt can inhibit Raf-1 activation through promoting the inhibitory iMST2/iRaf-1 complex formation and thus, Raf-1 activation was reduced with increasing Akt amount. On the other hand, at higher concentrations Akt phosphorylates and inhibits MST2 activity toward LATS1, which releases the LATS1 inhibition of Raf-1 through the feedback loop and thereby increasing ERK activity. Therefore, we asked how breaking the Ras/LATS1 balance may influence the response pattern of ERK. Interestingly, graded suppression of Ras activity transforms ERK activity from a concave-up biphasic to a monotonic decreasing pattern to a concave-down biphasic pattern (Figure 5e). Exactly the opposite observation was made when we strengthened the LATS1 feedback loop (Figure 5f). Taken together, these results suggest that which function Akt plays in regulating ERK activity is critically dependent on the balance between Ras and LATS1.

The last pattern found for Akt as the controlling protein was M1E1 in which Akt consistently inhibits both ERK and MST2 activation (Figure 5g). In this case, the protein interaction module plays a major role in driving these effects, as MST2-Raf-1 binding acts a strong negative regulator of ERK activation while the effect from LATS1 feedback and Ras are both relatively weak (Figure 5h). Indeed, blocking the LATS1 feedback (and Ras activation) did not really affect the network responses (Figure 5i and Supplementary Materials Figure S1b). An intuitive explanation is that increasing Akt depletes active MST2, thereby weakening the LATS feedback. Concurrently, accumulated inactive MST2 sequesters Raf-1 through their binding, leading to downregulated Raf-1 and thus ERK activity (Figure 5h).

### 3.4. Ras Displays Multi-Functional Role and Drives Biphasic Responses of both MST2 and ERK

**Figure 6:**
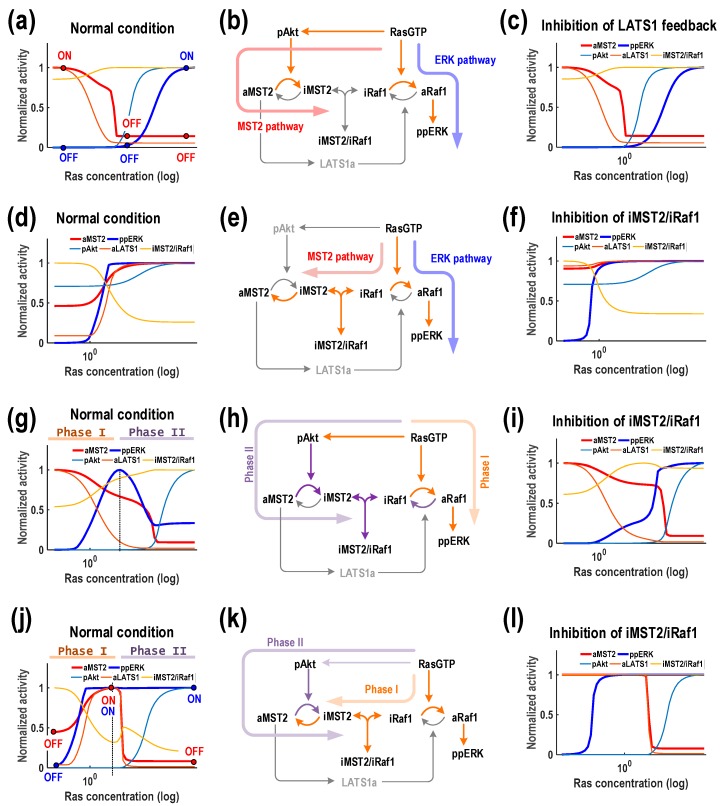
Ras displays multi-functional role. (**a**–**c**) The pattern M_1_E_2_ where increasing Ras robustly downregulates MST2 activity but increases ERK activity; (**d**–**f**) The pattern M_2_E_2_ where Ras triggers biphasic response profiles in ERK and MST2; respectively (**g**–**i**) The pattern M_1_E_3_ where increasing Ras downregulates ERK activity and induces a biphasic (concave-down) response of ERK activity; (**j**–**l**) The pattern M_3_E_2_ where Ras provokes a biphasic response for MST2 and increasing response for ERK. Note that the response profiles to Ras were generated using a representative kinetic parameter set that show the same patterns as in Figure 4 (see Supplementary Materials Table S4).

Unlike Akt, our simulations revealed that Ras can generate multiple response patterns for both MST2 and ERK activities. Specifically, Ras induces four different patterns, M_1_E_2_, M_1_E_3_, M_2_E_2_ and M_3_E_2_, showing that it can either promote or inhibit MST2 and ERK under specific conditions, and even display both roles in some cases (Figure 4, the second column). Since Ras is directly upstream of Raf-1, it is natural to expect increasing Ras abundance leads to increased ERK activity, and this was indeed seen in 3 observed patterns (M_1_E_2_, M_2_E_2_ and M_3_E_2_) which together account for the majority of the total simulated parameter sets (96%). What was not expected is that the Ras-induced MST2 activity profile is distinct in each case. We first consider pattern M_1_E_2_ where increasing Ras robustly downregulates MST2 activity (Figure 6a). This downregulation can be attributed to the link between Ras and Akt, as Ras promotes Akt activity to inhibit MST2. We observed that the switches regarding MST2 (“off”) and ERK activity (“on”) occurred at different Ras thresholds, which interestingly suggests that depending on Ras concentration, the system can turn ON only MST2 (low Ras), only ERK (high Ras), or shut down both (intermediate Ras). Ras thus serves multi-functional effect depending on its abundance. To evaluate whether the MST2/Raf-1 interaction module and/or the LATS1 feedback contribute to induction of the M_1_E_2_ pattern, we simulated the effect of blocking either the formation of Raf-1/MST2 complexes or the LATS1 feedback loop and found that these did not really influence response patterns of the system (Figure 6c and Supplementary Materials Figure S2a). This suggests that the M_1_E_2_ pattern emerged primarily through the Ras/Akt link but not the protein interaction module nor the LATS1 mediated feedback mechanism (Figure 6b).

Ras, in contrast, promotes MST2 signalling in M2E2 (Figure 6d). To tease out possible mechanisms governing this rather counterintuitive monotonic increase of MST2, we first perturbed the LATS1 feedback loop, which showed that varying the strength of this feedback has no effect on MST2 activity (Supplementary Materials Figure S2b). Perturbation of the protein interaction module however dramatically abolished the steep Ras-dependent increase of MST2 activation, turning it into an almost constant response (Figure 6f). These simulations lead to an explanation that Ras induced activation of Raf-1 steers it away from the MST2/Raf-1 complexes, leaving more available MST2 free for dimerization, resulting in increased MST2 activity (Figure 6e). This is supported by the steep depletion of the MST2/Raf-1 complexes as Ras levels increases (Figure 6d). In contrast to M_1_E_2_, the Ras/Akt link is necessarily weak here, which may be the case in specific cell types. 

This leaves the remaining patterns M_1_E_3_ and M_3_E_2_, where Ras triggers biphasic response profiles in ERK and MST2, respectively (Figure 6g,j). Like M_1_E_2_, the decreasing pattern for MST2 in M_1_E_3_ is underlined by a strong Ras-to-Akt link (Figure 6g). However, the biphasic nature of ERK activity in this pattern was caused by presence of a strong protein interaction module featuring tight association between MST2 and Raf-1 (Figure 6h), which was lacking in M_1_E_2_. Indeed, relaxing this association completely eliminated ERK biphasic profile, while largely maintained other components’ behaviours (Figure 6i). Thus, the biphasic response of ERK can be explained by splitting it into 2 phases as depicted in Figure 6g: low Ras upregulates ERK (Phase I), but as Ras level continues to rise, Akt is activated leading to accumulated inactive MST2 which subsequently sequesters Raf-1 into the complexes and inhibits its activation (Phase II). Of note, blocking the LATS1 feedback did not affect the overall systems responses, implying that this feedback is not functional in this pattern (Supplementary Materials Figure S2c). Finally, we examine M_3_E_2_ where Ras provokes essentially opposite dynamic behaviours: a biphasic response for MST2 and increasing response for ERK. Under this regime, simulations showed that Ras can turn off (or on) both MST2 and ERK, or selectively only turn on ERK activity (Figure 6j). The MST2/Raf-1 interaction module was again found critical in generating a biphasic MST2, as a weakened interaction completely removed this pattern (Figure 6l). Note that blocking the LATS1 feedback did not affect the overall systems responses (Supplementary Materials Figure S2d). Intuitively, the increase in MST2 activity at low Ras is due to more MST2 being available for activation as Raf-1 is sequestered away from its complexes with MST2 by Ras (Phase I). As Ras level rises, Akt becomes activated and starts to suppress MST2 by promoting the complexes formation (Phase II, Figure 6k).

Taken together, the above analyses revealed Ras as a highly flexible and dynamic regulator of both MST2 and ERK activity. Although a Ras-induced biphasic response of MST2 seems to be a rare event (only 4% of the total simulations) compared to other patterns, it nevertheless could occur given the right conditions. Which role Ras exactly assumes is strongly dependent on its abundance in cells, and is not trivially recognised without detailed analysis of the network structure.

### 3.5. EGFR Generates Multi-Phasic ERK Response

We also examined possible dynamics generated by EGFR and detected only two different dynamic patterns (M_1_E_2_ and M_1_E_4_), in both cases MST2 showed a monotonic decrease trend against rising EGFR expression. ERK activity displayed a monotonic increase profile in M_1_E_2_, (accounting for most of the simulations at 85%) but showed a combined pattern of both type I and type II biphasic response in M1E4 (15%, Figure 4, right column).

**Figure 7:**
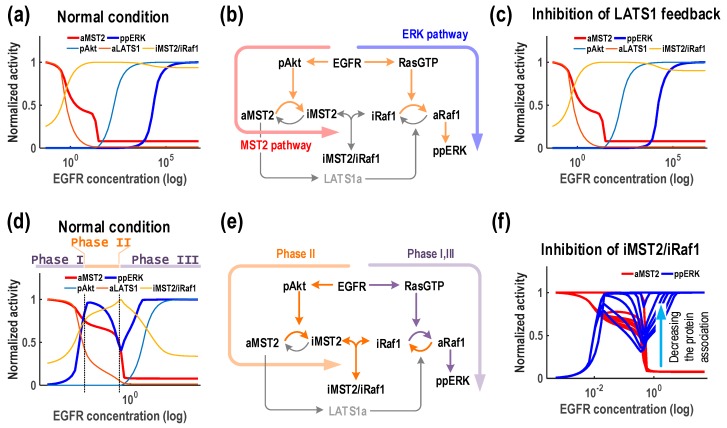
EGFR generates multi-phasic ERK response. (**a**–**c**) The pattern M_1_E_2_ where EGFR robustly downregulates MST2 activity but increases ERK activity; (**d**–**f**) The pattern M_1_E_4_ where the activation of ERK shows an initial increase (Phase I) at low EGFR, followed by a decrease (Phase II) at intermediate EGFR levels, but bounced back (Phase III) again at high EGFR levels. The association coefficient between Raf-1 and MST2 was gradually blocked and the temporal decrease pattern of the activation of ppERK disappeared accordingly (indicated by the arrow, right panel). Note that the response profiles to EGFR were generated using a representative parameter set that show the same patterns as in Figure 4 (see Supplementary Materials Table S4).

The M_1_E_2_ pattern and its explanation were similar to that induced by Ras (Figure 6a) since EGFR is direct upstream of Ras which can also directly promotes Akt signalling. M_1_E_4_ was more curious as the activation of ERK showed an initial increase (Phase I) at low EGFR, followed by a decrease (Phase II) at intermediate EGFR levels, but bounced back (Phase III) again at high EGFR (Figure 7d). ERK activation in Phase I can be explained by the direct activation of Raf-1/ERK via Ras. Phase II was due to accumulation of inactive MST2 by EGFR and Ras induced Akt, which binds to and inhibits Raf-1 and thus ERK. Phase III can be brought about by further activation of Ras that overrides the inhibitory effect of MST2 through the interaction with Raf-1. We confirmed this mechanism by blocking the protein association between Raf-1 and MST2 and found that the activation of ppERK did not display the temporal decrease pattern (Figure 7f).

## 4. Discussion and Conclusions

How biochemical signalling pathways process signals and specify biological responses has been a fundamental question in cell signalling and systems biology fields. As part of our efforts to elucidate Hippo signalling, we have recently linked the pro-apoptotic Hippo/MST2 pathway to the proliferative ERK pathway and deciphered the molecular basis that governs their interaction. This includes multiple mechanisms of cross-regulation involving the protein interaction module of MST2 and Raf-1 and the LATS1-to-Raf feedback regulation [6,7,30]. In addition, Akt was found to play a critical role in coordinating the opposing activities of the Hippo and ERK signalling axes [6,8]. Here, we hypothesize that the interplay of these regulatory mechanisms in combination with variation in the protein expression of the network components confer rich and diverse systems dynamics to the network in different cellular contexts. We set out to test this hypothesis and explore the context-specific dynamics of the network using computational modelling and analyses.

Our analyses revealed that the Hippo-ERK signalling network is capable of generating significantly rich and diverse nonlinear input-output behaviours depending on the cellular contexts, which could be clustered into several distinct patterns. We considered changes in the concentrations of Akt, Ras and EGFR as the key inputs, while the activity levels of MST2 and ERK as the major outputs that are indicative of the signalling readouts of the Hippo and ERK pathway, respectively. Our results confirmed the posed hypothesis that the involvement of various regulatory modules (including the Ras-to-Akt linkage, MST2-Raf-1 interaction module, and the LATS1-to-Raf feedback regulation) to different extents in combination with variations in the expression profiles of the network nodes generate the observed diverse response patterns of MST2 and ERK activation. Removal of the feedback loop from ERK to Raf-1 did not have significant effect on the steady-state solution and response profiles of MST2 and ERK activation in our parameter setting, indicating it was functionally weak in this context. However, this feedback may have more substantial role in different biological contexts [16,23]. Further focused simulations enabled us to provide mechanistic explanation that underlines the behaviour of each clustered pattern, illustrating how they could emerge at the molecular level and under which network contexts. Most interestingly, we discovered that Akt can play completely different functional roles towards ERK in a dose-dependent manner. Under certain network specification, Akt acts as a facilitator of ERK activity at low dose but as an inhibitor instead at high concentration. While Akt always inhibits MST2 activity, its dose-specific function towards ERK suggests that Akt has the capacity to independently tune a signalling branch without affecting the other. Such flexibility in network dynamics control brings robustness that enables the cells to utilise the same network architecture for different purposes. In addition, we also found Ras displays a multi-functional, dose-determined role and drives the biphasic responses of both MST2 and ERK activation. Interestingly, EGFR can even generate a multi-phasic ERK response.

Incoherent feed-forward regulation (IFF) is one of the most abundant network motifs found in biological systems [30,34] and a salient feature of its dynamics is characterised by a bi-phasic dependence of the regulated output on the level of the regulator (input). This biphasic dependence arises within the IFF motif due to the fact that the output node is both positively and negatively regulated by the input via different routes. In fact, biphasic responses observed in many biological systems have been attributed to IFF regulation [35]. Our results revealed that the Hippo-ERK network (shown in Figure 1) frequently displays biphasic behaviours (e.g., Akt induces biphasic (concave-down and -up) responses of ERK activity, and Ras induces biphasic responses of both ERK and MST2 activities) but interestingly it does not possess the IFF network motif explicitly. We found that in both cases, the Raf-1/MST2 interaction plays a critical role in causing the biphasic responses and its interplay with the LATS1 mediated feedback is necessary in the case of Akt as the controlling dose. These findings reinforce the emerging concept that protein-protein interaction motif can also induce effects of an incoherent feed-forward loop regulation when it is embedded into signalling network [7].

Although cells originating from different tissues or patients may harbour similar signalling network architecture, these networks may differ significantly in connectivity (edge) strength and/or protein (node) abundances [10,11,12]. Such variations are among the chief reasons anti-cancer targeted therapies are effective in some patients but not others; and often underline conflicting observations between different laboratories [19,36,37,38,39]. Understand exactly how these variations influence the dynamics of signalling networks in general, and the Hippo pathway in particular, is an imperative task that may help reconcile inconsistent experimental data. However, a comprehensive answer to this question is experimentally challenging, but feasible through computational modelling and analysis. In this study, kinetic modelling and model-based analysis have been successfully exploited to unveil the salient context-specific dynamic features of Hippo-ERK signalling. While many detected dynamic patterns of the network and the underlying molecular basis remain to be confirmed experimentally, our results provide a valuable foundation for future work by offering new systems-level insights into the complex dynamic behaviours of the Hippo and ERK signalling systems.

## Supplementary Materials

The following are available online at www.mdpi.com/2073-4425/7/8/44/s1, Figure S1: Various response patterns generated by increasing Akt, Figure S2: Various response patterns generated by increasing Ras, Table S1: Reactions and reaction rates of the Hippo-ERK network model, Table S2: Ordinary differential equations of the Hippo-ERK network model, Table S3: Parameter values used in the model, Table S4: Parameter values clustered for the response pattern classification.

## References

[1] Xin M., Kim Y., Sutherland L.B., Murakami M., Qi X., McAnally J., Porrello E.R., Mahmoud A.I., Tan W., Shelton J.M. (2013). Hippo pathway effector yap promotes cardiac regeneration. Proc. Natl. Acad. Sci. USA.

[2] Varelas X. (2014). The Hippo pathway effectors TAZ and YAP in development, homeostasis and disease. Development.

[3] Harvey K.F., Zhang X., Thomas D.M. (2013). The Hippo pathway and human cancer. Nat. Rev. Cancer.

[4] Hansen C.G., Moroishi T., Guan K.L. (2015). YAP and TAZ: A nexus for Hippo signaling and beyond. Trends Cell Biol..

[5] Piccolo S., Dupont S., Cordenonsi M. (2014). The biology of YAP/TAZ: Hippo signaling and beyond. Physiol. Rev..

[6] Romano D., Nguyen L.K., Matallanas D., Halasz M., Doherty C., Kholodenko B.N., Kolch W. (2014). Protein interaction switches coordinate Raf-1 and MST2/Hippo signalling. Nat. Cell Biol..

[7] Nguyen L.K., Matallanas D.G., Romano D., Kholodenko B.N., Kolch W. (2015). Competing to coordinate cell fate decisions: The MST2-Raf-1 signaling device. Cell Cycle.

[8] Romano D., Matallanas D., Weitsman G., Preisinger C., Ng T., Kolch W. (2010). Proapoptotic kinase MST2 coordinates signaling crosstalk between RASSF1a, Raf-1, and Akt. Cancer Res..

[9] Dhillon A.S., Meikle S., Yazici Z., Eulitz M., Kolch W. (2002). Regulation of Raf-1 activation and signalling by dephosphorylation. EMBO J..

[10] Geiger T., Wehner A., Schaab C., Cox J., Mann M. (2012). Comparative proteomic analysis of eleven common cell lines reveals ubiquitous but varying expression of most proteins. Mol. Cell. Proteom..

[11] Wu L., Candille S.I., Choi Y., Xie D., Jiang L., Li-Pook-Than J., Tang H., Snyder M. (2013). Variation and genetic control of protein abundance in humans. Nature.

[12] Zhang B., Wang J., Wang X., Zhu J., Liu Q., Shi Z., Chambers M.C., Zimmerman L.J., Shaddox K.F., Kim S. (2014). Proteogenomic characterization of human colon and rectal cancer. Nature.

[13] Von Kriegsheim A., Baiocchi D., Birtwistle M., Sumpton D., Bienvenut W., Morrice N., Yamada K., Lamond A., Kalna G., Orton R. (2009). Cell fate decisions are specified by the dynamic ERK interactome. Nat. Cell Biol..

[14] Suire S., Hawkins P., Stephens L. (2002). Activation of phosphoinositide 3-kinase γ by Ras. Curr. Biol..

[15] Borisov N., Aksamitiene E., Kiyatkin A., Legewie S., Berkhout J., Maiwald T., Kaimachnikov N.P., Timmer J., Hoek J.B., Kholodenko B.N. (2009). Systems-level interactions between insulin-EGF networks amplify mitogenic signaling. Mol. Syst. Biol..

[16] Shin S.Y., Rath O., Choo S.M., Fee F., McFerran B., Kolch W., Cho K.H. (2009). Positive- and negative-feedback regulations coordinate the dynamic behavior of the Ras-Raf-MEK-ERK signal transduction pathway. J. Cell Sci..

[17] Man K.F., Tang K.S., Kwong S. (1996). Genetic algorithms: Concepts and applications (in engineering design). IEEE Trans. Ind. Electron..

[18] Shin D., Kim I.S., Lee J.M., Shin S.Y., Lee J.H., Baek S.H., Cho K.H. (2014). The hidden switches underlying roralpha-mediated circuits that critically regulate uncontrolled cell proliferation. J. Mol. Cell Biol..

[19] Shin S.Y., Kim T., Lee H.S., Kang J.H., Lee J.Y., Cho K.H., Kim do H. (2014). The switching role of β-adrenergic receptor signalling in cell survival or death decision of cardiomyocytes. Nat. Commun..

[20] Srinivas M., Patnaik L.M. (1994). Genetic algorithms: A survey. Computer.

[21] Kholodenko B.N., Demin O.V., Moehren G., Hoek J.B. (1999). Quantification of short term signaling by the epidermal growth factor receptor. J. Biol. Chem..

[22] Shin S.Y., Rath O., Zebisch A., Choo S.M., Kolch W., Cho K.H. (2010). Functional roles of multiple feedback loops in extracellular signal-regulated kinase and wnt signaling pathways that regulate epithelial-mesenchymal transition. Cancer Res..

[23] Won J.K., Yang H.W., Shin S.Y., Lee J.H., Heo W.D., Cho K.H. (2012). The crossregulation between ERK and PI3K signaling pathways determines the tumoricidal efficacy of MEK inhibitor. J. Mol. Cell Biol..

[24] Byrne K.M., Monsefi N., Dawson J.C., Degasperi A., Bukowski-Wills J.C., Volinsky N., Dobrzynski M., Birtwistle M.R., Tsyganov M.A., Kiyatkin A. (2016). Bistability in the Rac1, PAK, and RhoA signaling network drives actin cytoskeleton dynamics and cell motility switches. Cell Syst..

[25] Nguyen L.K., Cavadas M.A., Scholz C.C., Fitzpatrick S.F., Bruning U., Cummins E.P., Tambuwala M.M., Manresa M.C., Kholodenko B.N., Taylor C.T. (2013). A dynamic model of the hypoxia-inducible factor 1alpha (HIF-1alpha) network. J. Cell Sci..

[26] Ferrell J.E. (2008). Feedback regulation of opposing enzymes generates robust, all-or-none bistable responses. Curr. Biol..

[27] Brandman O., Ferrell J.E., Li R., Meyer T. (2005). Interlinked fast and slow positive feedback loops drive reliable cell decisions. Science.

[28] Brandman O., Meyer T. (2008). Feedback loops shape cellular signals in space and time. Science.

[29] Pomerening J.R., Kim S.Y., Ferrell J.E. (2005). Systems-level dissection of the cell-cycle oscillator: Bypassing positive feedback produces damped oscillations. Cell.

[30] Varusai T.M., Kolch W., Kholodenko B.N., Nguyen L.K. (2015). Protein-protein interactions generate hidden feedback and feed-forward loops to trigger bistable switches, oscillations and biphasic dose-responses. Mol. BioSyst..

[31] Trewavas A. (2002). Plant cell signal transduction: The emerging phenotype. Plant Cell.

[32] Kholodenko B.N., Kolch W. (2008). Giving space to cell signaling. Cell.

[33] Kholodenko B.N., Hancock J.F., Kolch W. (2010). Signalling ballet in space and time. Nat. Rev. Mol. Cell Biol..

[34] Kim D., Kwon Y.K., Cho K.H. (2008). The biphasic behavior of incoherent feed-forward loops in biomolecular regulatory networks. Bioessays.

[35] Ma W., Trusina A., El-Samad H., Lim W.A., Tang C. (2009). Defining network topologies that can achieve biochemical adaptation. Cell.

[36] Shin S.-Y., Yang H.W., Kim J.-R., Do Heo W., Cho K.-H. (2011). A hidden incoherent switch regulates RCAN1 in the calcineurin-NFAT signaling network. J. Cell Sci..

[37] Shin S.Y., Yang J.M., Choo S.M., Kwon K.S., Cho K.H. (2008). System-level investigation into the regulatory mechanism of the calcineurin/NFAT signaling pathway. Cell. Signal..

[38] Sanna B., Brandt E.B., Kaiser R.A., Pfluger P., Witt S.A., Kimball T.R., van Rooij E., de Windt L.J., Rothenberg M.E., Tschop M.H. (2006). Modulatory calcineurin-interacting proteins 1 and 2 function as calcineurin facilitators in vivo. Proc. Natl. Acad. Sci. USA.

[39] Vega R.B., Rothermel B.A., Weinheimer C.J., Kovacs A., Naseem R.H., Bassel-Duby R., Williams R.S., Olson E.N. (2003). Dual roles of modulatory calcineurin-interacting protein 1 in cardiac hypertrophy. Proc. Natl. Acad. Sci. USA.

