# Supplementary material for: Unveiling Hidden Dynamics of Hippo Signalling: A Systems Analysis
Source: Genes (Basel). 2016 Aug 5;7(8):44. doi: 10.3390/genes7080044 (PMC4999832; doi:10.3390/genes7080044)
Supplement: Supplementary file 1 [file genes-07-00044-s001.docx]

Supplementary Materials: Unveiling Hidden Dynamics of Hippo Signalling: A Systems Analysis

Sung-Young Shin and Lan K. Nguyen

**Figure S1.** Various dynamics patterns generated by increasing Akt. (**a**) Inhibition of the LATS1 mediated feedback regulation replaces the biphasic response profile of ERK activity by a monotonic increasing pattern; (**b**) The response profile profiles are not affected by perturbation of Ras activity within 10-fold of the nominal value.

**Figure S2.** Various dynamics patterns generated by increasing Ras. (**a**) Blocking the formation of Raf-1/MST2 complexes did not influence systems dynamics; (**b**) Varying the strength of this feedback has no effect on MST2 and ERK activity; (**c**) Blocking the LATS1 feedback did not affect the overall systems dynamics; (**d**) The LATS1 feedback did not have an effect on the overall systems dynamics.

**Table S1.** Reactions and reaction rates of the Hippo-ERK network model.

| **Reaction rate** | **Reaction** | **Reaction Rates** |
| --- | --- | --- |
| v1 | Akt → pAkt | kc11*aEGFR*Akt/(Km11 + Akt) |
| v2 | pAkt → Akt | V11*pAkt/(Km12 + pAkt) |
| v3 | Akt → pAkt | kc12*Akt*RasGTP/(Km13 + Akt) |
| v4 | aMST2 → MST2 | V21*aMST2/(Km21 + aMST2) |
| v5 | iMST2 → MST2 | V22*iMST2/(Km22 + iMST2) |
| v6 | MST2 → dMST2 | ka21*MST2^2^ |
| v7 | MST2 + RASSF1A  ↔ MST2uRASSF1A | ka22*MST2*RASSF1A—kd21*MST2uRASSF1A |
| v8 | MST2 → iMST2 | kc21*MST2*pAkt/(Km23 + MST2) |
| v9 | dMST2 → aMST2 | kd31*dMST2 |
| v10 | aMST2 + RASSF1A  ↔ aMST2uRASSF1A | ka41*aMST2*RASSF1A—kd41*aMST2uRASSF1A |
| v11 | MST2uRASSF1A  → aMST2uRASSF1A | V51*MST2uRASSF1A/(Km51 + MST2uRASSF1A) |
| v12 | iMST2 + iRaf1  ↔ iRaf1uiMST2 | ka71*iMST2*iRaf1—kd71*iRaf1uiMST2 |
| v13 | aLATS1 → LATS1 | V81*aLATS1/(Km81 + aLATS1) |
| v14 | LATS1 → aLATS1 | kc81*aMST2*LATS1/(Km82 + LATS1) |
| v15 | LATS1 → aLATS1 | kc82*aMST2uRASSF1A*LATS1/(Km83 + LATS1) |
| v16 | Raf1 → iRaf1 | V91*Raf1/(Km91 + Raf1) |
| v17 | Raf1 → iRaf1 | kc91*aLATS1*Raf1/(Km92 + Raf1) |
| v18 | iRaf1 → Raf1 | kc92*iRaf1*RasGTP/(Km93 + iRaf1) |
| v19 | aRaf1 → Raf1 | V101*aRaf1/(Km101 + aRaf1) |
| v20 | Raf1 → aRaf1 | V102*Raf1/(Km102 + Raf1) |
| v21 | ipRaf1→ aRaf1 | V111*ipRaf1/(Km111 + ipRaf1) |
| v22 | aRaf1 → ipRaf1 | kc112*aRaf1*ppERK/(Km112 + aRaf1) |
| v23 | RasGDP → RasGTP | kc121*aEGFR*RasGDP/(Km121 + RasGDP) |
| v24 | RasGTP → RasGDP | V121*RasGTP/(Km122 + RasGTP) |
| v25 | ERK → ppERK | kc131*aRaf1*ERK/(Km131 + ERK) |
| v26 | ppERK → ERK | V131*ppERK/(Km132 + ppERK) |

**Table S2.** Ordinary differential equations of the Hippo-ERK network model. The reaction rates are given in Supplementary Table 1.

| **Left-Had Sides** | **Right-Hand Sides** | **Initial Conditions (nM)** |
| --- | --- | --- |
| d[Akt]/dt | −v1 + v2 − v3 | 200 |
| d[pAkt]/dt | v1 − v2 + v3 | 0 |
| d[MST2]/dt | v4 + v5 − 2 × v6 − v7 − v8 | 0.17 |
| d[dMST2]/dt | −v9 + v6 | 209.9 |
| d[aMST2]/dt | −v4 − v10 + 2 × v9 | 94.83 |
| d[aMST2uRASSF1A]/dt | v10 + v11 | 483.73 |
| d[MST2uRASSF1A]/dt | v7 − v11 | 1.51 |
| d[RASSF1A]/dt | −v7 − v10 | 14.76 |
| d[iMST2]/dt | −v5 + v8 − v12 | 0 |
| d[iRaf1uiMST2]/dt | v12 | 200 |
| d[aLATS1]/dt | −v13 + v14 + v15 | 184.7 |
| d[LATS1]/dt | v13 − v14 − v15 | 15.31 |
| d[iRaf1]/dt | −v12 + v16 + v17 − v18 | 750 |
| d[Raf1]/dt | −v16 − v17 + v18 + v19 − v20 | 0 |
| d[aRaf1]/dt | −v19 + v20 + v21 − v22 | 0 |
| d[ipRaf1]/dt | −v21 + v22 | 0 |
| d[RasGDP]/dt | −v23 + v24 | 300 |
| d[RasGTP]/dt | v23 − v24 | 0 |
| d[ERK]/dt | −v25 + v26 | 3000 |
| d[ppERK]/dt | v25 − v26 | 0 |

**Table S3.** Parameter values used in the model.

| **Parameter** | **Value** | **Unit** | **Parameter** | **Value** | **Unit** |
| --- | --- | --- | --- | --- | --- |
| kc121 | 2.061 × 10^−1^ | min^−1^ | Km121 | 1.205 × 10^2^ | nM |
| V121 | 1.027 × 10^3^ | nM·min^−1^ | Km122 | 2.972 × 10^2^ | nM |
| kc11 | 1.149 × 10^−3^ | min^−1^ | Km11 | 5.121 × 10^1^ | nM |
| kc12 | 7.170 × 10^−1^ | min^−1^ | Km13 | 7.440 × 10^−1^ | nM |
| V11 | 8.687 × 10^−2^ | nM·min^−1^ | Km12 | 1.497 × 10^−2^ | nM |
| kc92 | 9.203 × 10^−1^ | min^−1^ | Km93 | 9.015 × 10^−1^ | nM |
| V91 | 2.071 × 10^0^ | nM·min^−1^ | Km91 | 8.821 × 10^−1^ | nM |
| V102 | 3.173 × 10^2^ | nM·min^−1^ | Km102 | 3.197 × 10^0^ | nM |
| V101 | 9.948 × 10^2^ | nM·min^−1^ | Km101 | 4.575 × 10^2^ | nM |
| kc131 | 5.342 × 10^0^ | min^−1^ | Km131 | 3.676 × 10^−2^ | nM |
| V131 | 9.953 × 10^2^ | nM·min^−1^ | Km92 | 1.068 × 10^1^ | nM |
| Km132 | 1.510 × 10^2^ | nM | kc21 | 6.684 × 10^3^ | min^−1^ |
| Km23 | 8.313 × 10^−4^ | nM | V22 | 7.511 × 10^3^ | nM·min^−1^ |
| Km22 | 8.162 × 10^2^ | nM | ka71 | 2.812 × 10^1^ | nM^−1^·min^−1^ |
| kd71 | 4.886 × 10^−4^ | min^−1^ | ka21 | 4.472 × 10^3^ | nM^−1^·min^−1^ |
| kd31 | 6.117 × 10^−1^ | min^−1^ | V21 | 1.414 × 10^3^ | nM·min^−1^ |
| Km21 | 4.273 × 10^2^ | nM | ka22 | 6.840 × 10^−2^ | nM^−1^·min^−1^ |
| kd21 | 1.130 × 10^−1^ | min^−1^ | ka41 | 4.237 × 10^−1^ | nM^−1^·min^−1^ |
| kd41 | 1.226 × 10^0^ | min^−1^ | V51 | 5.688 × 10^−4^ | nM·min^−1^ |
| Km51 | 6.708 × 10^0^ | nM | kc81 | 6.189 × 10^3^ | nM^−1^·min^−1^ |
| Km82 | 3.961 × 10^3^ | nM | kc82 | 2.930 × 10^−4^ | nM^−1^·min^−1^ |
| Km83 | 2.226 × 10^1^ | nM | V81 | 2.261 × 10^3^ | nM·min^−1^ |
| Km81 | 8.503 × 10^−2^ | nM | kc112 | 2.742 × 10^−3^ | nM^−1^·min^−1^ |
| Km112 | 2.071 × 10^2^ | nM | V111 | 2.547 × 10^2^ | nM·min^−1^ |
| Km111 | 7.678 × 10^−2^ | nM | kc91 | 1.177 × 10^−1^ | nM^−1^·min^−1^ |

**Table S4.** Representative parameter values that were used for generation of Figures 5–7.

| **Controlling Protein** | **Pattern** | **Kinetic Parameter** | | | | | |
| --- | --- | --- | --- | --- | --- | --- | --- |
|  |  | **kc121** | **kc11** | **kc12** | **kc92** | **kc112** | **kc91** |
| **Akt** | M_1_E_1_ | 7.596 × 10^−2^ | 6.196 × 10^0^ | 4.526 × 10^−2^ | 3.162 × 10^−1^ | 9.482 × 10^−1^ | 1.893 × 10^−1^ |
|  | M_1_E_3_ | 5.075 × 10^−3^ | 2.746 × 10^−2^ | 5.193 × 10^−3^ | 3.574 × 10^0^ | 1.020 × 10^0^ | 1.075 × 10^2^ |
|  | M_1_E_4_ | 2.287 × 10^1^ | 3.697 × 10^2^ | 2.440 × 10^−2^ | 7.172 × 10^2^ | 1.177 × 10^2^ | 4.829 × 10^2^ |
| **Ras** | M_1_E_2_ | 4.370 × 10^−1^ | 9.701 × 10^−3^ | 1.301 × 10^0^ | 4.771 × 10^−1^ | 7.904 × 10^−1^ | 9.969 × 10^−1^ |
|  | M_1_E_3_ | 5.545 × 10^−1^ | 3.035 × 10^−3^ | 7.936 × 10^−3^ | 1.096 × 10^2^ | 1.773 × 10^0^ | 2.577 × 10^2^ |
|  | M_2_E_2_ | 6.317 × 10^−2^ | 2.458 × 10^−2^ | 2.831 × 10^−3^ | 4.235 × 10^2^ | 1.995 × 10^2^ | 3.627 × 10^−2^ |
|  | M_3_E_2_ | 9.313 × 10^2^ | 5.661 × 10^−2^ | 3.568 × 10^−3^ | 1.357 × 10^2^ | 7.658 × 10^2^ | 2.081 × 10^−3^ |
| **EGFR** | M_1_E_2_ | 1.917 × 10^−1^ | 2.249 × 10^0^ | 2.722 × 10^−1^ | 6.282 × 10^−1^ | 1.363 × 10^0^ | 1.595 × 10^0^ |
|  | M_1_E_4_ | 1.775 × 10^2^ | 2.248 × 10^−2^ | 3.325 × 10^−2^ | 3.196 × 10^2^ | 5.945 × 10^2^ | 4.639 × 10^0^ |
